# Multifaceted roles of *IKZF1* gene, perspectives from bench to bedside

**DOI:** 10.3389/fonc.2024.1383419

**Published:** 2024-06-24

**Authors:** Lin Feng, Hang Zhang, Ting Liu

**Affiliations:** Department of Hematology, Institute of Hematology, West China Hospital of Sichuan University, Chengdu, Sichuan, China

**Keywords:** *IKZF1* gene, IKAROS, BCP-ALL, Ph+ALL, transcription factor, MRD

## Abstract

The *IKZF1* gene encodes a transcription factor that belongs to the family of zinc-finger DNA-binding proteins associated with chromatin remodeling. The protein product, IKAROS, had been proved to regulate lymphopoiesis. Subsequent mouse model studies have further confirmed its regulating role in lymphopoiesis as well as in hematopoiesis; besides, it associates with immune function, certain immune disorders like common variable immunodeficiency and dysgammaglobulinemia have been proved to be associated with germline *IKZF1* mutations. Dysfunction of IKAROS also bears paramount significance in leukemic transformation and alterations of *IKZF1* gene predicts a poor prognosis in hematological malignancies. As an independent prognostic marker, *IKZF1* has been incorporated in the risk stratification of BCP-ALL and stratification-guided therapy has also been generated. In this review, we provide a concise and comprehensive overview on the multifaceted roles of *IKZF1* gene.

## Introduction

**Figure 1:**
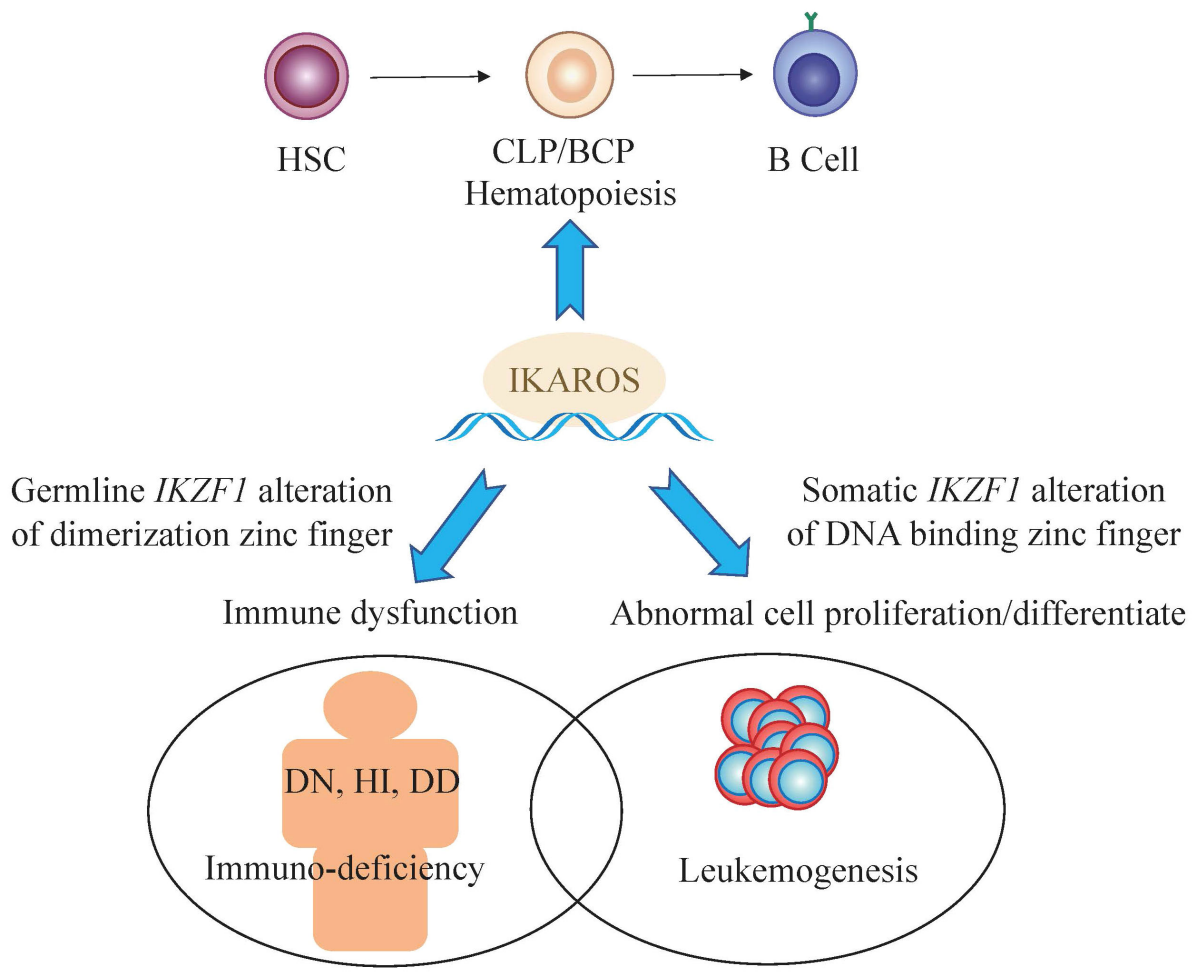
The multifaceted roles of *IKZF1.* * HSC, hematopoietic stem cell; CLP, common lymphoid precursor; BCP, B cell progenitor; DN, Dominant negative effect on IKAROS function; HI, Haploinsufficiency due to quantitative or qualitative decrease of IKAROS; DD, Dimerization defects caused by deletion of dimerization zinc fingers.

*IKZF1*, namely LyF-1, had been proved to play an important role for the first time in lymphopoiesis (1). IKAROS as a founding member of a family of zinc finger transcription factors, encoded by the *IKZF1* gene, associates with other zinc finger bearing transcription factors (HELIOS, AIOLOS, EOS, and PEGASUS encoded by *IKZF2*, *IKZF3*, *IKZF4* and *IKZF5*, respectively) in the regulation of both myeloid and erythroid lineage, in addition to lymphoid lineage (2–9). Germline or somatic mutations of *IKZF1* demonstrate quite variable clinical phenotypes, ranging from primary immunodeficiency (PID)/inborn errors of immunity (IEI) (10–12) to autoimmune diseases (13–16) and to even hematological malignancies such as acute lymphoblastic leukemia (17, 18). As in acute lymphoblastic leukemia, especially of high-risk B-cell-precursor ALL (BCP-ALL), in which *IKZF1* gene mutation is of high frequency (19, 20), such as *BCR-ABL* positive ALL and *BCR-ABL*-like ALL, genetic alteration of *IKZF1* gene is associated of poor outcome (21–23) and confers resistance in conventional chemotherapy (24, 25). In terms of the types of mutations in the *IKZF1* gene, the most common are characterized by large fragment deletions mapping to *IKZF1* exons, as well as by other point mutations (missense, nonsense) or frameshift mutation, which cause diversity in clinical phenotypes (**Figure 1**).

## Structure and function of *IKZF1* gene

From early 1990s, the studies *in vitro* and *in vivo* revealed the molecular structure of *IKZF1* gene, followed by precise elaboration of the functional domains of its corresponding protein IKAROS (1, 26). The *IKZF1* gene is mapped on chromosome 7 at 7p12.2 and consists of 8 exons, coding for 519 amino acids (27). It is worth noting that expression of *IKZF1* gene is restricted to the fetal and adult hemo-lymphopoietic system, and IKAROS is one of the most important and founding member of a family of zinc finger transcription factors, which associates with other transcription factors, HELIOS, AIOLOS, EOS, and PEGASUS, in regulation of hemopoiesis.

**Figure 2:**
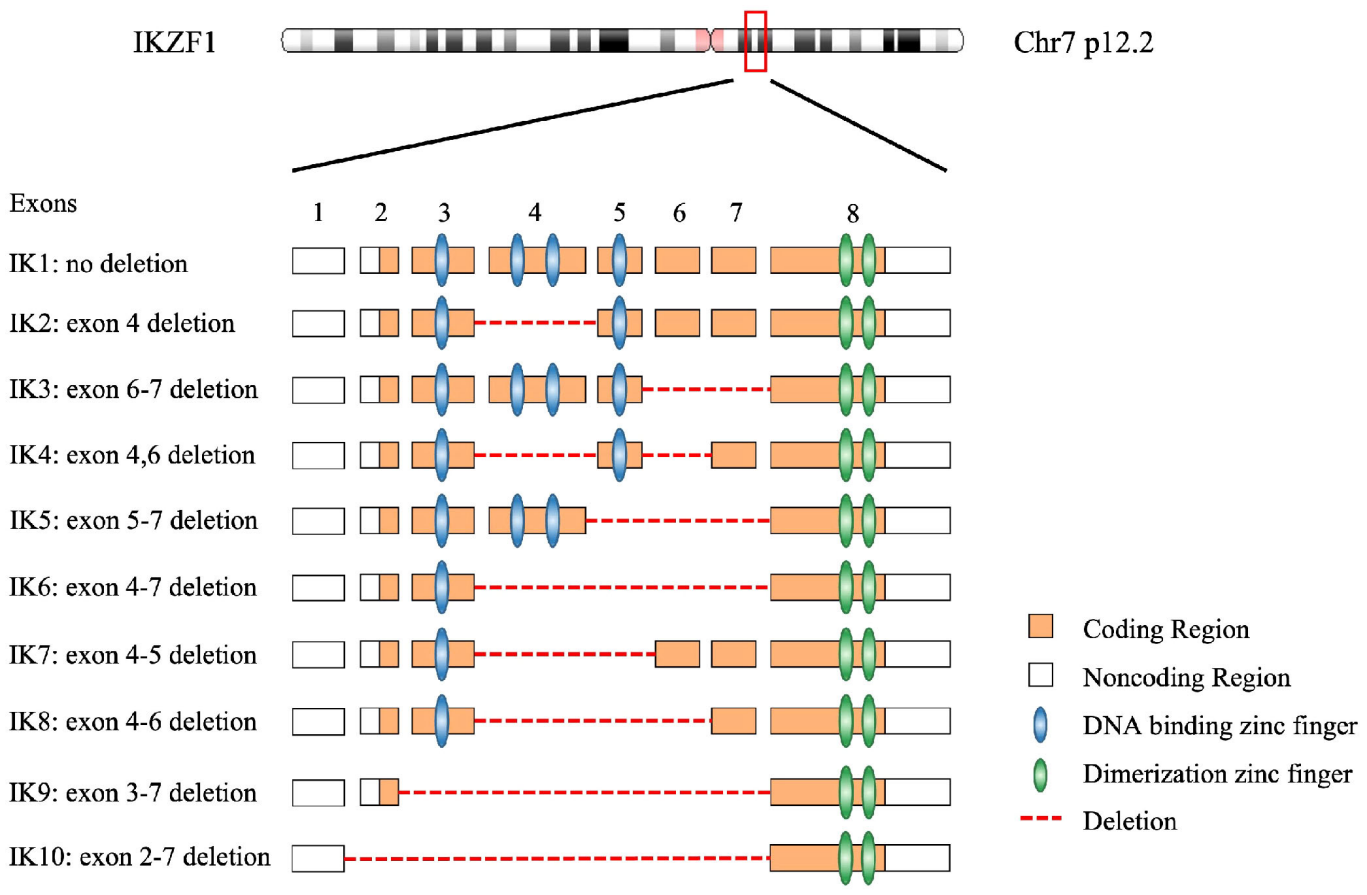
The genome of *IKZF1* and most common 10 IKAROS isoforms with their functional domains. A total of 18 isoforms have been described on NCBI database.

IKAROS functions as a master transcription factor and is characterized by the DNA-binding ability through its zinc-finger domains to regulate target genes involved in hematopoiesis, particularly in lymphocyte differentiation, through association with transcriptional complexes or transcription factors including the nucleosome remodeling and deacetylase (NuRD) complex and epigenetic modification interferon regulatory factor 4 (IRF4) (28–30). Among these zinc-finger domains, four zinc-fingers (ZF1–4), coding by *IKZF1* exons 4–6, are located at the N-terminus of the protein and bind to the targeted DNA sequences when the protein is phosphorylated and being subsequently transferred from the cell cytoplasm to nucleus to exerts its function (31, 32). Homo- or heterodimerization of wild type IKAROS with other IKAROS family members facilitate the localization of the dimers to the pericentromeric heterochromatin (PC-HC) and regulates the expression of its target genes (33, 34). *IKZF1* mutations which locate in the DNA-binding domains would cause compromised DNA-binding ability of mutant IKAROS and demonstrate a bizarre staining pattern of PC-HC (35). While, the rest of the zinc-fingers (ZF5–6), being coded by exon 8 of *IKZF1*, are in the location of the C-terminus of the protein, required for homo- and heterodimerization between the different IKAROS proteins (26, 36). Dimerization between IKAROS zinc-finger proteins with a functional DNA binding domain facilitate their DNA-binding ability and transcriptional activity. The mutations affecting zinc-finger 5 and/or zinc-finger 6 would limit homo-and heterodimerization, in addition to a compromised DNA-binding ability and abnormal PC-HC localization (17). According to NCBI database (27), there are at least 18 isoforms of *IKZF1*, which are generated by alternative splicing transcript variants. The isoforms share the C-terminal dimerization zinc-fingers in common, and have variant numbers and locations of DNA binding zinc-fingers in the N-terminal (26) (**Figure 2**).

Among all IKAROS isoforms, IK6 (Δ4–7) is the most common, with a proportion of up to 42%, followed by IK10 (Δ2–7) ranging from 7% to 20%, which is consistent in adult and pediatric patients (21–23). The remaining approximately one third are comprised of the so-called rare isoforms because frequency of each of these variants is no more than 10% in BCP-ALL patients, such as Δ2–3, Δ2–7, Δ2–8, etc. (**Table 1**). Interestingly, in terms of prognosis, there seems to be no significant difference between IK6 and IK10 isoforms (21), possibly because IK6 and IK10 share the similar structure that both isoforms lack all four N-terminal zinc-finger containing domains, thus generating a similar clinical phenotype. Nevertheless, almost all isoforms due to various *IKZF1* deletions confer a poor prognosis including those rare IKAROS isoforms.

**Table 1:** Percentages of common *IKZF1* isoforms in Pediatric and Adult BCP-ALL.

IKAROS isoforms	Pediatric (%)	Adult (%)
Δ4–7 (IK6)	36.4	42
Δ2–7 (IK10)	13.6	7–20
Δ2–8	4.5	~10
Δ4–8	9.1	~10

*Δ: large fragment deletions mapping to IKZF1 gene exons.

## Roles of *IKZF1* gene in hematopoiesis

**Figure 3:**
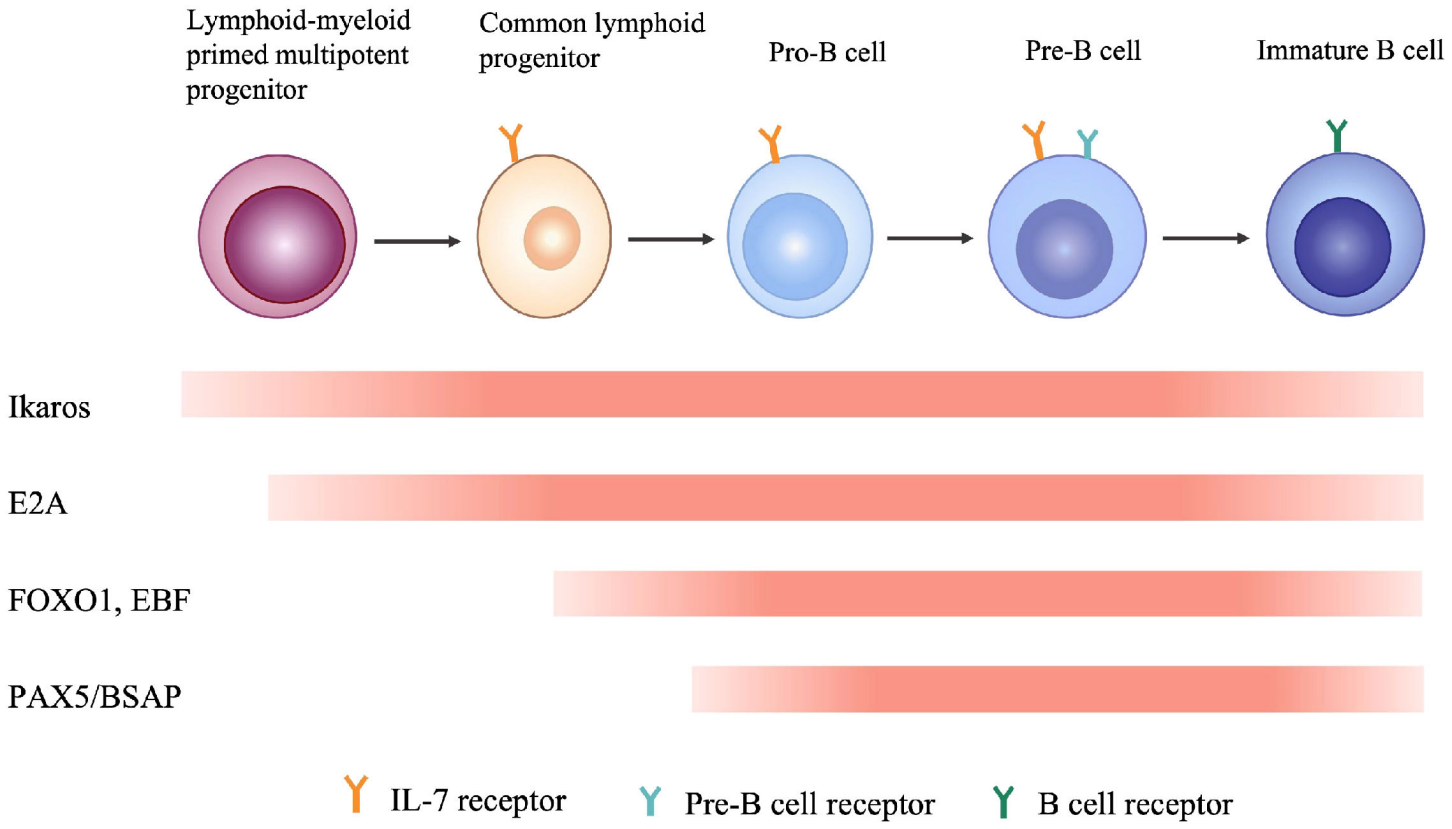
Multiple transcription factors are involved in different stages of B cell development in bone marrow.

B-cell and T-cell lineages develop from an uncommitted hematopoietic stem cell. The somatic gene rearrangements that generate the highly diverse repertoire of antigen receptors, immunoglobulin for B cells, and the T-cell receptor for T cells, occur in the early stages of the development of T cells and B cells from a common bone marrow–derived lymphoid progenitor. During the development of these lymphoid cells, a large amount of transcription factors cooperates and play vital roles in the process. The IKAROS functions as a master transcription regulator in the differentiation of lymphoid cells. Unlike its transcription factor counterparts such as EA2, FOXO1, EBF and PAX5/BASP, whose expression persists only in certain stages of B cell development in the bone marrow, *IKZF1* is expressed throughout the whole process from lymphoid-myeloid primed multipotent progenitor (LMPP) to immature B cell within the bone marrow (37–39) (**Figure 3**). Transcription factors abnormalities could be harmful to the cell homeostasis and mediate leukemic transformation (40). There is a high frequency of transcription factors abnormalities in B-cell precursor lymphoblastic leukemia (BCP-ALL) and are recognized as the main genetic-hit events in the leukemogenesis (41). Mouse model studies by Kirstetter P et al. showed that IKAROS activity fluctuations due to various types of *IKZF1* mutation had huge impact on multiple aspects of B cell lineage development (42).

Other mouse model studies by Winandy S et al. have evidenced that IKAROS is required for both early and late stage of lymphocyte differentiation in the thymus. IKAROS dysfunction will enhance the proliferation of maturing thymocytes and eventually lead to malignant transformation of such thymocytes (6, 43). Furthermore, Georgopoulos K et al. demonstrated that mice harboring homozygous germline mutation of *IKZF1* were lack of not only adaptive immune lymphocytes of T and B cells, but innate immune lymphocytes of natural killer cells, in addition to deficiency of their corresponding progenitor cells, yet with preservation of normal erythroid and myeloid cells (6, 44).

However, Lopez R et al. showed in their mouse model studies a different phenotype that the IKAROS null mice had anemia and megakaryocytic abnormalities, besides lymphoid and stem cell defects (45). One possible explanation for this divergent manifestation could be the variant location of the lost zinc-fingers between the two studies. In the studies of Georgopoulos K et al., the lost zinc fingers located in the N-terminal domains for DNA-binding, which leads to the truncate IKAROS protein. Lopez R et al. deleted the entire C-terminal zinc fingers required for IKAROS protein dimerization and function, the loss of C-terminal zinc fingers probably interferes with the normal PYR complex formation on DNA (44, 46), followed by the subsequent development block of whole blood lineages. In consistent with studies by Lopez R et al., Francis O et al. and Malinge S et al. also found that IKAROS functioned as a regulator of myeloid and erythroid differentiation (7–9).

## Roles of *IKZF1* mutations in immune function

During the whole 1990s and early 2000s, researches on IKAROS mainly focus on the its role in regulation of hematopoiesis, especially lymphopoiesis. In the new era, more studies are carried out, striving to elaborate the correlation between *IKZF1* mutation and the resultant impact on immune function. As described above in mouse models that *IKZF1* null mice are prone to T-cell malignancies like T-ALL or lymphoma, nevertheless in the real world, humans harboring germline mutation of *IKZF1* are more frequently linked to various types of immunodeficiency instead of malignancies (10, 11, 14–16, 47–53).

**Figure 4:**
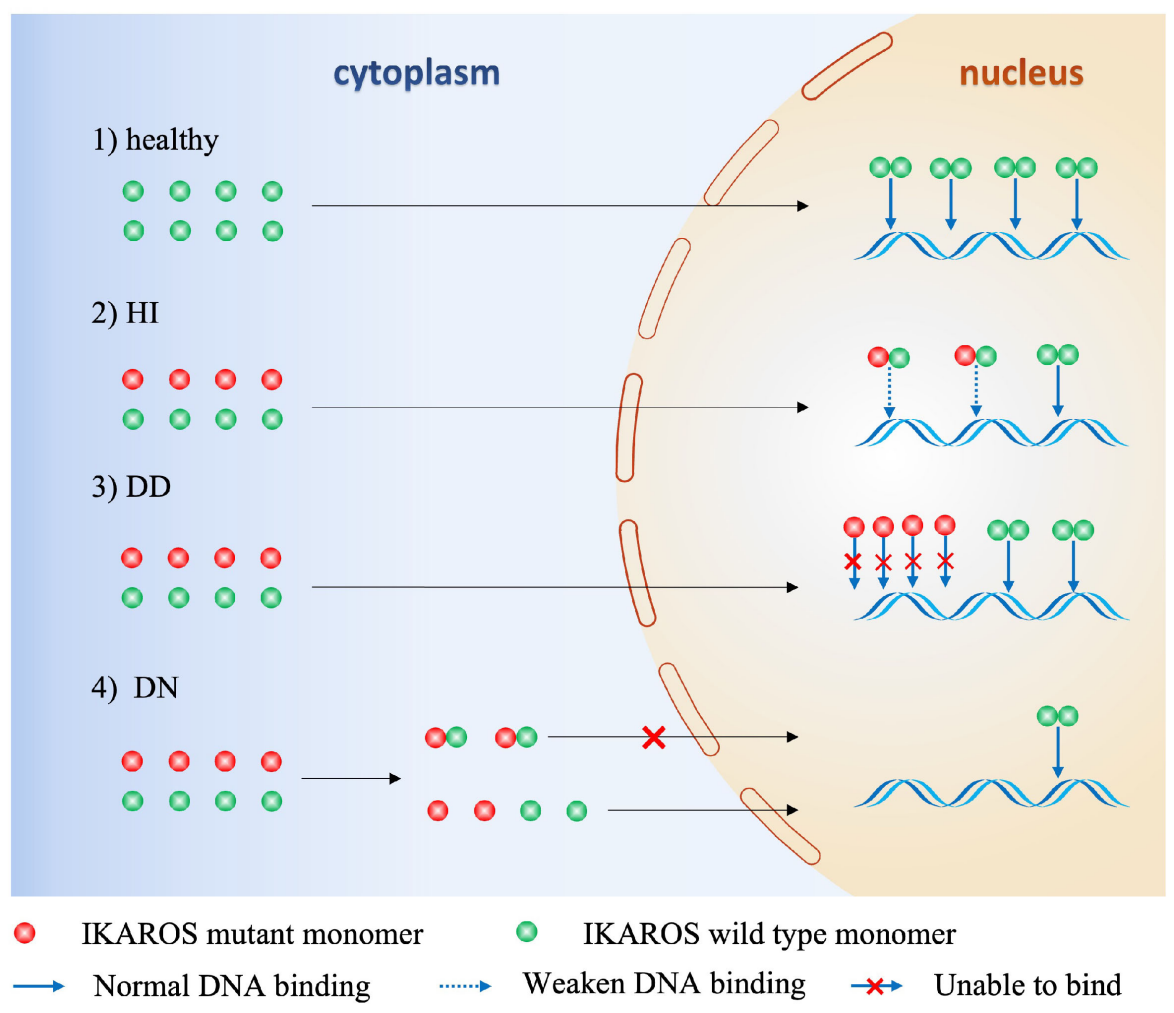
Mechanisms of functional defects of IKAROS caused by germline alterations of *IKZF1*. HI, Haploinsufficiency due to quantitative or qualitative decrease of IKAROS, without influencing the wild-type IKAROS. DD, Dimerization defects caused by deletion of dimerization zinc fingers lead to more monomers that cannot bind to DNA sequence, without influencing the wild-type IKAROS. DN, Dominant negative effect on IKAROS function. The mutant monomer can combine with wild-type monomer, but the dimer lacks DNA-binding or PC-HC targeting function, thus affecting the function of the wild-type IKAROS.

Studies from mouse models and humans suggest that there is strong correlation between genotype of the numerous variants of *IKZF1* gene and phenotype of the clinical patients. The key factor defining the phenotype is the protein expression of IKAROS which is dependent on the germline alterations of *IKZF1* gene, and according to these genetic and proteic variations, three clinical groups are established among those primary immunodeficiencies/inborn errors of immunity patients (12): Haploinsufficiency (HI), Dimerization defective (DD) and Dominant negative (DN). 1). HI due to quantitative or qualitive decrease of IKAROS: Large deletions of IKZF1 exons or nonsense mutation leading to impaired protein expression results in reduction of wild type IKAROS expression, or IKAROS function loss resulting from disruption of DNA-binding and PC-HC targeting, yet without influencing the wild type IKAROS protein; 2) DD due to genetic alterations (ZF5–6 involved) affecting IKAROS dimerization ability: In contrast to loss of ability to interact with IKAROS family members and generate homo- or heterodimers, DD mutants conserve the DNA-binding ability as monomers and have no impact on wild type IKAROS; 3) DN: One special IKAROS mutants that conserve the dimerization ability but are unable to bind IKAROS target sequences and disrupt interaction between PC-HC and wild type IKAROS proteins (**Figure 4**). In terms of IKAROS dosage effect, theoretically speaking, DN type has the most adverse impact on the normal immune function, for one thing mutants of DN type exhibit HI effect, for another DN mutants hijack the wild type IKAROS protein to worsen the normal IKAROS functional defects. Boutboul D et al. evidenced that patients with DN mutations would probably have an earlier onset of combined immunodeficiency compared to patients harboring HI or DD mutations, in addition, patients with DN mutations had a more severe loss of Pan-B cells ranging from progenitor B cells to antibody-secreting plasma cells (47). Winandy S et al. and Georgopoulos K et al. also showed in their studies that normal hemo-lymphopoiesis was more strongly influenced among DN genotype mouse who were devoid of both innate immune NK cells and adaptive immune T, B cells and the corresponding progenitor cells (6, 44). Other studies found that nearly all patients with DN mutations had low numbers of B cells in the peripheral blood in contrast to a significant lower prevalence of decreased PB B cells in patients with HI and DD mutations (17, 37, 38, 48, 51). Additionally, patients with DN mutations had more naïve T cells (CD45RA+CD62L+ or CD45RA+CD45RO–) and an abnormal ratio of Th subsets of which both CD4+ memory T cells and Treg cells were decreased, owing to impaired sensitivity to IL-2 stimulation, reduced STAT5 pathway activation (47). As one of professional antigen-presenting cells, dendritic cells (DC) play important roles not just in the initiation and activation of adaptive immune response but also in the process of T cell maturation in the thymus when early T-cell precursor cells (ETPs) differentiate from double-negative stage to single-positive naïve T cell (37). Mouse model and human studies also showed that DN genotype had lower numbers of DC (5, 44, 54). Monocytes and neutrophils are classical phagocytes responsible for defensing against invading bacteria and other microbes. Patients with DN mutations had reduced granulopoiesis of neutrophils and were incapable of full responses to stimulations to Toll-like receptors and the subsequent scant production of proinflammatory cytokines by monocytes. It is rational that DN genotype would confer a more severe clinical phenotype, in consistent with the idea, Thaventhiran J et al. and other researchers found that patients with DN mutations were susceptible to an earlier onset and broader as well as more severe invasive infections, including pneumocystis pneumonia which was infrequent in patients harboring HI or DD mutations (53, 55). Although DN genotype often implies an inferior phenotype, patients with DN mutations would barely have chances of developing autoimmune diseases like systemic lupus erythematosus (SLE), immune thrombocytopenia (ITP), rheumatoid arthritis (RA), antiphospholipid syndrome (APS) and others, of which are more frequently seen in DD patients followed by HI patients (13, 15, 16, 49–51, 56). Unlike DN genotype which is characterized by devoid of B cells, allelic variants of DD and HI are more likely to generate self-reactive B cells due to incapable of controlling B cell anergy and TLR signaling (57). As clearly described in Janeway’s immunobiology textbook, IKAROS is a rare transcription factor that expressed throughout the B cell development in the bone marrow and regulates many vital events like maintaining a suitable intensity of TLR signaling and induction of B cell anergy, which contribute to B cell central tolerance (37). Furthermore, Kuehn H et al. showed that DD mutants had an abnormal sumoylation and affected the normal interaction between IKAROS and NuRD complex, leading to aberrant epigenetic modification and the consequent B cell central intolerance which counts for much in the pathogenesis of autoimmune diseases (17).

## Roles of *IKZF1* mutations in leukemia

### Frequency of *IKZF1* alterations in leukemia

Most of our knowledge of T-cell development in the thymus comes from the mouse, experiments on mice demonstrates that T-cell precursors migrate from the bone marrow to the thymus, where they commit to the T-cell lineage after Notch receptor signaling. Unlike those transcription factors (TCF1, GATA3, Bcl 11b) expressed at a later stage of T-cell development in the thymus (37), Georgopoulos K et al. found that *IKZF1* gene was expressed throughout the ontogeny of the T cell lineage from as early in the hemopoietic progenitors in the fetal stage to the adult stage of T cell maturation (1). Soon afterwards, further findings from IKAROS heterozygous mice studies by Winandy S et al. showed that compromised IKAROS function leads to aberrant proliferation of clonal T cells and the consequent development of T cell leukemia and lymphoma (6). There is a strong link between IKAROS inactivation and Notch activation in murine T-ALL, so it is rationale to deduce that IKAROS defect could promote Notch activation in human T-ALL. However, *IKZF1* is rarely found in human T-ALL (<5%) (23, 58–60). The patients with high Notch signaling expression appear to express normal IKAROS, except for the most aggressive T-ALL subtype, ETP-ALL, in which the frequency of *IKZF1* somatic mutations is 13% (61). It is also similar in terms of acute myeloid leukemia (AML), the frequency of *IKZF1* somatic mutations is only 3.83% (62). Interestingly, Grossmann V et al. found that chronic myeloid leukemia (CML) patients had a slightly higher mutation rate of *IKZF1*(17.9%) when progressing to blast crisis (63). Jäger R et al. also showed that transformed MPN patients had a higher incidence of heterozygous loss of *IKZF1* compared to that in non-leukemic MPN patients (21% vs 0.2%) (64). In sharp contrast to the low frequency among the aforementioned hematological malignancies, *IKZF1* alterations is of much higher frequency in BCP-ALL (19, 22), particularly in kinase and cytokine- receptor signaling activating leukemias including Ph+ALL and Ph-like ALL (59, 60, 65–68). Despite a high incidence of *IKZF1* mutations in BCP-ALL, these alterations are mainly linked to somatic abnormalities, which are not characterized by point mutation (missense, nonsense) or frameshift mutation, but by large fragment deletions mapping to *IKZF1* exons (21–23). Churchman M et al. showed that germline *IKZF1* alterations is extremely low in pediatric BCP-ALL (69).

#### *IKZF1* alterations promote leukemogenesis and induction of chemo-resistance

IKZF1 alterations promote leukemogenesis had been studied in mouse and human by Churchman M et al. and other researchers (22, 70, 71). *IKZF1* alterations conferred stem cell-like properties characterized by overexpression of genes indicative of a stem cell phenotype and lead to upregulation of adhesion molecules (FAK, RHO, CTNND1, etc.) as well as integrins, altogether facilitating self-renewal or leukemic transformation. Furthermore, as a master regulator of B cell differentiation, functional impairment of IKAROS due to *IKZF1* somatic alterations would affect its binding to IgH locus and disturb arrangement of the heavy chain of pre-BCR and BCR, thus signaling through the pre-BCR that mediates the transition from pre-B cell to immature B cell would be blocked (42). Besides, downstream of pre-BCR signaling is impacted where expression of c-Myc is upregulated and k-light chain is downregulated which enhance the cell cycle in pro-B and pre-B cells, and block their differentiation into immature B cells (72–74). Li JF et al. reported that IKZF1 N159Y is a rare subtype of B-ALL characterized by significant upregulation of the transcriptional coactivator YAP1, SALL1 and ARHGEF28B, and downregulation of the B-cell receptor signaling and JAK-STAT signaling pathways (75).

In addition, *IKZF1* alterations also make BCP-ALL, in particular high-risk subtype such as Ph+ALL and Ph-like ALL, refractory to treatment by induction of chemo-resistance through various mechanisms. Marke R et al. demonstrated in their mouse models studies and *in vitro* experiments that compromised functional IKAROS due to *IKZF1* alterations affected the transcription function of TSC22D3, DUSP1, IRS2, and so on, combined with the abnormal regulation of GC-activated genes in response to prednisolone, contributing to resistance to GC-induced apoptosis (23). Scheijen B et al. showed that *IKZF1* haplodeficient B-cells enhanced cell survival as compared to wild-type (WT) IKAROS, when co-existing with BTG1 deficiency, these B-cells showed an even stronger GC-induced apoptosis (76). Of note, under physiological conditions, WT IKAROS controls the transition of pre-B cells from a stroma-adherent proliferative phase to a relative non-dividing but differentiating phase. As evidenced by Joshi I et al. and Churchman M et al. (77, 78), IKZF1 alterations resulted in overexpression of adhesion molecules and upregulation of focal adhesion kinase (FAK). The dysregulated FAK pathway synergizing with other upregulated intracellular signaling pathways promoted the survival of leukemic cells. Furthermore, *IKZF1* deletions that co-occurred with deletions in *CDKN2A*, *CDKN2B*, *PAX5*, or *PAR1* in the absence of *ERG* deletion conferred the worst outcome and, consequently, were grouped as *IKZF1* ^plus^ (79).

#### *IKZF1* alterations and hereditary leukemia

Germline mutations have been known for many years that they could increase risks of various cancers, as TP53 germline mutation could increase risks of acute lymphoblastic leukemia. Until 2018, Churchman et al. demonstrated in their study that germline variants in *IKZF1* predispose to ALL (69). Winer P et al. also reported that *IKZF1* germline mutation contribute to acute lymphoblastic leukemia in children with Down syndrome (DS) (80). By germline whole-exome sequencing (WES) study, they discovered pathogenic variant p.Arg162Trp in *IKZF1* gene to ALL predisposition, which was also reported in sporadic B-ALL patients (69). Buitenkamp T et al. showed that DS-ALL patients had a similar frequency of *IKZF1* deletions to that of high-risk (HR) non-DS-ALL patients (~30%) (81). Palmi C et al. discovered that the *IKZF1* ^plus^ feature in DS-ALL was 3 times more frequent than in non-DS-ALL (18% versus 6%), and *IKZF1* ^plus^ patients had even worse outcome as well as higher relapse (82). Furthermore, they also found that the blasts of *IKZF1* ^plus^ DS-ALL patients were particularly sensitive to drugs which had been described to be effective in Ph-like cases, such as HDAC inhibitors. These findings could help refine stratification-directed therapy for DS-ALL patients, who are intrinskly more fragile and are of inferior outcome compared with non-DS children with ALL.

#### *IKZF1* deletion is of high prevalence and a pivotal prognostic factor in BCP-ALL

As early as 2007, a large cohort study enrolling 1522 adult patients of ALL including Ph- and Ph+ ALL from multicenter demonstrated that karyotype could be an independent prognostic factor in adult ALL (83), however, karyotype itself has limited potency to stratify patients to the ideal risk groups and ensure that patients received the most appropriate treatment. Acute lymphoblastic leukemia patients can only benefit the most from the pretreatment evaluation of combing high quality cytogenetic data with precise molecular genetic analysis. So, we can see that during the recent two decades, extensive researches have been done trying to find out the optimal combination of the methods, and *IKZF1* alterations are just one of the most important constituents to this combination. As described above, *IKZF1* is a master regulator of hematopoiesis, especially lymphopoiesis. Loss of function of IKAROS due to *IKZF1* alterations causes a predisposition to lymphoid malignancies. Mullighan C et al. and others reported that *IKZF1* alterations is frequent in BCP-ALL, about 15% in pediatric patients and increasing to 30%-50% in adult patients, and remarkably higher in high-risk subtypes, notably, the frequency is as high as 84% and 70% in adult Ph+ALL and Ph-like ALL, respectively (19, 22, 59, 60, 65–68, 84, 85).

Either the initial study from Mullighan C (22) in which 258 ALL pediatric patients were enrolled or the following numerous large scale clinical researches (67, 86–90) which included more than 1000 pediatric patients and the relatively small-scale adult clinical researches (91, 92), all showed that *IKZF1* deletion was a strong prognostic factor and confer an increased risk of relapse and poor outcome with inferior EFS as well as OS in BCP-ALL. This is in consistence with our studies (93). Furthermore, *IKZF1* deletion demonstrates heterogeneous impact on the prognosis of BCP-ALL patients when coexisting with other molecular abnormalities. Ribera J et al. and others (94, 95) reported that patients with coexistence of *IKZF1* and *CDKN2A/B* alterations had worse prognosis. Stanulla M et al. and Zaliova M et al. also pointed out that patients harboring *IKZF1* ^plus^ alterations had the worst prognosis compared to those with sole *IKZF1* deletion (79, 96). The patients with *IKZF1* ^plus^, that have co-occurring deletions in *CDKN2A*, *CDKN2B*, *PAX5*, or *PAR1*, worsen the B-ALL phenotype, as the co-aberrations enhance B cell development arrest and abnormal proliferation. It is of great interest that BCP-ALL pediatric patients with *ERG* deletions had a high co-occurring rate of *IKZF1* deletions, and such patients confer a good outcome compared to patients with the sole-*IKZF1* deletions or the sole-*EGR* deletions (97, 98). Ultimately, the prognostic significance of *IKZF1* deletions translate into optimal therapeutic strategies to benefit patients from unnecessary treatment related toxicity and ensure sufficient treatment intensity to avoid chemo-resistance or relapse. It is noteworthy that combing MRD and *IKZF1* status could refine the risk stratification and better predict the disease relapse (99, 100).

#### *IKZF1* mutations in acute myeloid leukemia

The role and implications of IKZF1 mutations and deletions are well studied in B-ALL. However, the prevalence and impact in AML remain sparse. Eckardt J et al. reported the 2.8% of patients harboring *IKZF1* alterations in 1606 adult AML patients, and the heterozygous SNVs are the most common mode of alteration. They also identified a mutational hotspot in the second N-terminal zinc finger domain at p.N159S, which was present in 19 of 45 (42.2%) *IKZF1* alterations cases. AML patients with mutated *IKZF1* was usually associated with aberration in *RUNX1*, *GATA2*, *KRAS*, *KIT*, *SF3B1*, and *ETV6*. *IKZF1* mutation could be an independent marker of adverse risk regarding complete remission rate, event-free, relapse-free, and overall survival (101). Zhang X et al. investigated 522 newly diagnosed AML patients, 20 of whom harboring *IKZF1* mutations with a significant co-occurrence of mutations in *SF3B1*, *CSF3R*, and *CEBPA*. The authors describe a significantly reduced CR rate for patients with *IKZF1* mutations (62). Wang Y et al. found that 23 out of 475 (4.8%) AML patients bear mutated *IKZF1*, and delineated three clusters of *IKZF*-mutated as: N159S (40%), co-occurring CEBPA mutations (43%), and others (17%) (102). Jäger et al. found deletions of *IKZF1* to occur in ~20% of AML cases that arose secondary to myeloproliferative neoplasms suggesting a differential role of deletions and mutations in myeloid leukemogenesis (64). Intriguingly, *IKZF2*, which encodes another member of IKZF family of transcription factors (HELIOS) also has a role in AML, as shown in the study by Park S et al. (103), *IKZF2* has an oncogenic role in AML, and *IKZF2* is required for leukemic stem cells survival and function by controlling chromatin accessibility of self-renewal and differentiation programs.

## Treatment of BCP-ALL patients target to transcriptional regulation of genes by IKAROS

As reviewed above, *IKZF1* alterations promote leukemogenesis, it seems quite rational to restore wild type *IKZF1* expression in the treatment of BCP-ALL with *IKZF1* alterations. Mullighan C et al. pioneered this work and showed that retinoid receptor agonists could induce expression of wild-type *IKZF1*, reversed the stem cell features of *IKZF1*-altered leukemic cells and increased responsiveness to dasatinib in Ph+ ALL (104). Song C et al. demonstrated that TBB and CX-4945, different kinds of casein kinase II inhibitors, restored IKAROS activity and showed an antileukemia effect by inhibiting the transcription of the genes involved in the PI3K pathway (105). Palmi C et al. studied some compounds *in vitro* cell culture that show one of histone deacetylase inhibitors, Givinostat, has the highest efficacy on *IKZF1* ^plus^ blasts in comparison to controls (82). In the clinical setting, the direct targeting of IKZF1 to restore its function have not been yet. These studies suggested that targeting IKAROS pathways could be used as a therapeutic approach for the high-risk BCP-ALL in the future.

## Conclusions and perspectives

In conclusion, it has been clearly demonstrated that *IKZF1* alterations have great impacts on many aspects. Germline or somatic alterations as well as heterozygous or homozygous deletions, each exhibits a heterogeneous clinical phenotype. As is evidenced by mouse model and human studies that germline *IKZF1* alterations are highly associated with immune dysfunction including various types of immunodeficiency, in addition to autoimmune diseases; while for somatic deletions of *IKZF1*, patients are susceptible to acute B cell lymphoblastic leukemia. The roles of *IKZF1* alterations in leukemogenesis and potency in prognosis prediction have been elucidating. In the future, targeting IKAROS-regulated signaling pathways could be a highly effectively therapeutic approach. Identification of IKAROS target genes would be much important, both to gain insight into IKAROS to function as a tumor suppressor, as well as to identify novel therapeutic targets for the high-risk acute lymphoblastic leukemia.

## Author contributions

LF: Conceptualization, Writing – review & editing, Data curation, Formal analysis, Methodology. HZ: Data curation, Formal analysis, Writing – original draft. TL: Conceptualization, Funding acquisition, Writing – review & editing.
